# Benefits and obstacles of telemetric ICP monitoring

**DOI:** 10.1007/s00701-021-04730-5

**Published:** 2021-02-07

**Authors:** Joachim M. K. Oertel, Matthias J. M. Huelser

**Affiliations:** Department of Neurosurgery, Saarland University Medical Center and Saarland University Faculty of Medicine, Homburg, Saarland Germany

The commercial use of telemetric intracranial pressure (ICP) monitoring has been in use for over a decade now. The first device which was licensed is the Neurovent-P-tel probe® (Raumedic, Helmbrecht, Germany) in 2009 followed by reports of its first clinical use [2, 3, 8, 24]. Preclinical studies showed a sufficient long-term stability of the ICP measurement with only a minimal zero drift with good accuracy [9, 11, 12].

The second device followed with the Sensor Reservoir® which was approved for clinical use in 2015 (Christoph Miethke GmbH & Co.KG, Potsdam, Germany). The Sensor Reservoir® is to be integrated into the shunt system, and there is no limitation about the approved implantation time in opposition to the P-tel probe whose approved implantation time is limited to 90 days. The first experience with its clinical use was published in 2017 [6], and the first larger study appeared in an article in 2018 describing the experience of one of its biggest technical potentials—the intracranial pressure–guided shunt valve adjustment [1].

Nowadays, telemetric ICP monitoring is feasible and useable for a wide spectrum of pathologies.

First of all, compared to conventional ICP monitoring methods, it has the advantage of being a closed system decreasing the risk of infection [3, 13]. This is particularly an argument for using the P-tel device for ICP monitoring in the neuro-intensive care setting which is usually the application field for cabled ICP probes [15, 21].

In addition, the closed system and the long capable implantation duration facilitate different areas of application.

Long-term ICP monitoring with the Neurovent-P-tel® after ETV for 8 to 12 weeks is especially useful for treatment response prediction since the average time of ETV failure occurs within this time period [4, 10, 23].

Furthermore, it is a helpful tool for the diagnosis and prediction of shunt responsiveness in patients with complex hydrocephalus and ICP-related diseases, like idiopathic intracranial hypertension—in both adults and children [2, 14, 19, 20].

Additionally, it allows the monitoring of the shunt effect and the verification of a proper shunt function over a longer time period in complex shunt patients [1, 2, 19, 20].

Beyond that, the possibility of doing measurements in supine and vertical positions and over several measurement periods provides a clear distinction between over- and underdrainage. The potential of ICP-guided optimization of valve setting can be exploited to full extent due to use of both adjustable differential and anti-gravitational valves [1, 8, 18–20], which even a reduction in surgical revisions and radiation exposure due to a reduced necessity of imaging can be achieved [19]. Also, it provides a lower rate of hospitalization and there is even an alternative for home-telemonitoring [17, 19, 22].

“Does it change management” ask the authors of the study presented in the following, where a series of twelve shunt treated patients before and after insertion of a Sensor Reservoir® were investigated. Without giving away too much of the answer provided by this study, the authors found an improvement of symptoms in 75% of patients, a reduction of radiation exposure and hospitalization, and increased cost-effectiveness, similar to results which previous studies have yielded [1, 5, 19].

But telemetric ICP measurement also has its limitations and disadvantages.

First of all, the sampling frequency is significantly lower compared to cable bound devices (Shunt Reservoir® 40 Hz, Neurovent-P-tel® 5Hz). A single pressure curve analysis is therefore not possible, although measurement of pulse pressure amplitude with both devices is feasible. A further obstacle is the risk of zero drifting which obviously threatens the accuracy of the results. This problem occurs not only in telemetric ICP monitoring but also in conventional measurement. Nevertheless, because of the long durability of the implant, the time-dependent risk is higher in telemetric than in conventional measurements. The median shift from the baseline was in a study with P-tel® devices 2.5 mmHg on average (implantation time was often longer than the CE-approval time of 90 days) [16]. Therefore, analyzing dynamic ICP values like vasogenic slow waves and the pulse pressure amplitude is much more essential than static ICP values like the mean ICP [7]. Gathering this information is feasible with both devices but its analysis is not automatically done and therefore depends on the experience of the neurosurgeon and is time consuming. And in the case of the Shunt Sensor®, there is so far a lack of an appropriate analysis program. Here is room for improvement.

Nevertheless, telemetric ICP monitoring is already a valuable tool. The choice of the most suitable device depends on diagnostic goal in the individual case: for long-duration measurements, for example, for diagnostic purposes or for monitoring response to ETV, the Neurovent-P-tel® is to be preferred. For continuous, repeated ambulatory follow-up measurements intending to verify subtle drainage-related shunt failure and to control the valve setting adjustment in sequential outpatient presentations over years the Shunt Sensor® is more suitable.
